# Long-Term Success of Dental Implants in Atrophic Maxillae: A 3-Year Case Series Using Hydroxyapatite and L-PRF

**DOI:** 10.3390/bioengineering11121207

**Published:** 2024-11-28

**Authors:** Marco Andre Lomba Alves, Jakson Both, Carlos Fernando Mourão, Bruna Ghiraldini, Fabio Bezerra, Jose Mauro Granjeiro, Suelen Cristina Sartoretto, Monica Diuana Calasans-Maia

**Affiliations:** 1 Post-Graduation Program in Dentistry, Fluminense Federal University, Niteroi 24220-140, Brazil; dr.marcolomba@gmail.com; 2 Independent Researcher, Dois Vizinhos 85660-000, Brazil; both@wln.com.br; 3 Department of Periodontology, School of Dental Medicine Tufts University, Boston, MA 02111, USA; carlos.mourao@tufts.edu; 4 Dental Research Division, Dentistry School, Universidade Paulista, São Paulo 04710-000, Brazil; brunaguiraldini@yahoo.com.br; 5 Oral Surgery and Periodontology Department, São Paulo University, Ribeirão Preto 14040-904, Brazil; fabiobezerra@cenior.com.br; 6 Clinical Research Laboratory, Dentistry School, Universidade Federal Fluminense, Niteroi 24220-140, Brazil; jmgranjeiro@gmail.com (J.M.G.); susartoretto@hotmail.com (S.C.S.); 7 National Institute of Metrology, Quality and Technology (INMETRO), Duque de Caxias 25250-020, Brazil; 8 Oral Surgery Department, Fluminense Federal University, Niteroi 24220-140, Brazil

**Keywords:** hydroxyapatite, L-PRF, atrophic maxilla, dental implants, case series

## Abstract

Dental implants are essential for the prosthetic rehabilitation of edentulous patients, requiring adequate bone volume and density for osseointegration and load support. The posterior region of the maxilla, commonly deficient in bone quality and quantity, represents a clinical challenge. This case series reports an analysis involving 69 dental implants in the atrophic maxilla of nine patients. The procedures adopted combined alloplastic hydroxyapatite grafting and leukocyte platelet-rich fibrin (L-PRF) applied to the alveolar ridge and maxillary sinus lift. With an average follow up of three years after the installation of the prostheses, an implant success rate of 98.5% was observed, showing integration and functional stability. The strategy of combining hydroxyapatite with L-PRF proved to be effective in increasing bone volume and promoting osseointegration. These findings indicate that the technique and biomaterials are viable for rehabilitating atrophic maxillae in the posterior region, offering long-lasting clinical results and a high success rate.

## 1. Introduction

The predictability of dental implants as a treatment option for dental edentulism has been established over the past few decades. However, bone loss following edentulism can compromise implant placement, impacting prosthetic rehabilitation and patients’ quality of life. In such cases, restoring adequate three-dimensional bone volume is essential, particularly in the posterior maxilla, where bone density is often suboptimal [1]. Procedures such as horizontal and vertical bone augmentation, bone block grafting, and bilateral sinus lifts with subantral grafting have been widely employed to address significant bone deficiencies. Alternatives, such as zygomatic and short implants, have also been proposed to reduce the need for extensive bone augmentation procedures.

Bone grafts used in implantology to augment atrophic jaws can be classified by their origin into autogenous, allogenous, xenogenous, and alloplastic types. Autogenous grafts are considered the gold standard due to their biological mechanisms of osteogenesis, osteoconduction, and osteoinduction. However, their use is often limited by donor site morbidity and the finite amount of harvestable bone [2,3]. Allogenic grafts, sourced from individuals of the same species, eliminate the need for a secondary surgical site but may induce immune responses and rely on the availability of bone banks [4,5]. Xenogeneic grafts, derived from animal sources, offer advantages such as availability and ease of handling, though they may encounter barriers due to host immune responses and cultural considerations [6].

Alloplastic or synthetic grafts, such as calcium phosphate ceramics, including hydroxyapatite (HA), are widely used because of their biocompatibility and ability to serve as scaffolds for tissue repair [7,8]. Hydroxyapatite closely resembles the mineral component of bone, providing a biocompatible and osteoconductive scaffold that is particularly suitable for bone augmentation procedures [9].

L-PRF (leukocyte platelet-rich fibrin), derived from the patient’s blood, is rich in growth factors, leukocytes, and platelets and has been shown to enhance wound healing and tissue regeneration [10], offering complementary benefits, with HA providing structural support and L-PRF stimulating biological activity, which can significantly improve outcomes in reconstructive surgeries [11].

Despite the increasing use of synthetic HA and L-PRF, clinical data on their combined application for horizontal and vertical bone augmentation in severely atrophic maxillae remain limited. This clinical series aims to evaluate the effectiveness of combining synthetic HA with L-PRF in promoting bone gain and implant survival. The findings are expected to provide valuable insights for optimizing grafting protocols and enhancing prosthetic rehabilitation techniques for patients with maxillary atrophy.

## 2. Materials and Methods

This case series evaluated the survival of nine patients’ 69 dental implants placed in the atrophic maxillae. The treatment combined an alloplastic hydroxyapatite (HA) graft (Alobone^®^ Poros, Osseocon, Rio de Janeiro, Brazil) with leukocyte platelet-rich fibrin (L-PRF) and included an average clinical follow-up period of three years post-prosthesis installation. This study adhered to the PROCESS 2020 checklist to ensure transparency.

### 2.1. Biomaterials

#### 2.1.1. Physical and Chemical Characterization of the Biomaterial

The biomaterial used in this study underwent physicochemical characterization at the Biomaterials Laboratory (LABIOMAT) of the Brazilian Center for Physics Research (CBPF). Surface morphology was analyzed using scanning electron microscopy (SEM Quanta 250 FEI Company, Eindhoven, The Netherlands). Chemical groups present in the biomaterial were identified through Fourier-transform infrared spectroscopy (FTIR-IR, Prestige 21, Schimadzu, Kyoto, Japan). X-ray diffraction, XRD (ZEISS HZG4 high-resolution diffractometer, Carl Zeiss Jane Co., Jena, Germany) was employed to determine the crystalline mineral phases in the samples.

#### 2.1.2. Obtaining and Processing L-PRF

Peripheral blood was initially collected in two sterile 10 mL red-cap tubes and two sterile 10 mL white-cap tubes (BD Vacutainer^®^, Becton Serum Blood Collection Tubes, Dickinson & Company, Franklin Lakes, NJ, USA), all without anticoagulant, at a room temperature of 20 °C. L-PRF membranes were first prepared using the red-cap tubes according to the manufacturer’s protocol, which involved centrifugation at 2700 rpm for 12 min (~708 g) in a fixed/vertical angle centrifuge (IntraSpin™, Biohorizons^®^, Birmingham, Alabama, AL, USA). This protocol referenced the g-force value at the bottom of the centrifuge tubes.

**Figure 1:**
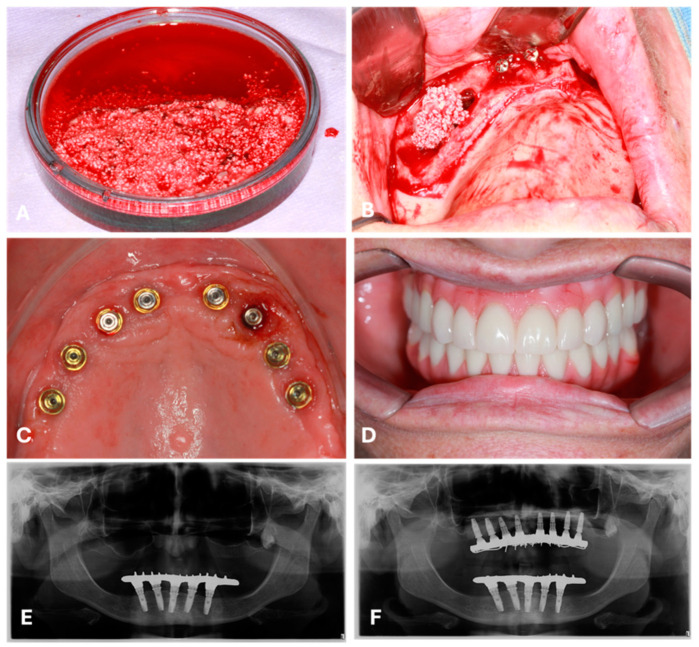
(**A**) Biomaterial for grafting: L-PRF combined with porous synthetic hydroxyapatite granules. (**B**) Lateral wall of the maxillary sinus exposed for grafting with the biomaterial. (**C**) Implant placement: eight implants installed five months post-graft surgery. (**D**) Rehabilitation: fixed prostheses placed four months after implant installation. (**E**,**F**) Panoramic radiographs showing maxillary atrophy (**E**) and bone restoration with implant placement following grafting (**F**).

After centrifugation, each L-PRF membrane was carefully removed from its tube and separated from the red phase at the base using sterile cotton tweezers. The yellow phase was then cut into small pieces and combined with synthetic hydroxyapatite granules. Subsequently, the two white-cap tubes were centrifuged at 700 rpm for 4 min to obtain the liquid phase of PRF. This liquid phase was incorporated into the initial mixture, which was left to agglutinate for 15 min at room temperature (Figure 1A).

### 2.2. Patients

This study was conducted following the Declaration of Helsinki, and informed consent was obtained from all subjects involved in this study.

This study included patients with severe atrophic maxillae who were rehabilitated with fixed protocol prostheses (ceramic or acrylic) supported by implants placed in grafted areas. All procedures were performed at a private clinic (Both Instituto de Odontologia, Dois Vizinhos, Brazil) by the same dental surgeon responsible for the clinic (J.B.). Inclusion criteria required healthy adult patients (ASA I) and non-smokers with total maxillary edentulism and a residual alveolar bone crest at least 4 mm thick and 2 mm high in the posterior maxilla.

Treatment followed a two-stage surgical approach. The first involved bone reconstruction, including vertical augmentation with bilateral sinus lifting and subantral grafting (Figure 1B), and horizontal augmentation using a mixture of L-PRF and synthetic hydroxyapatite. The second stage consisted of implant placement (Figure 1C), with a minimum healing period of five months between surgeries.

The prostheses were made using a delayed loading protocol after the implants had healed for four months (Figure 1D). Individualized studies and surgical planning were carried out for all cases using panoramic X-rays from the face (Figure 1E,F).

### 2.3. Surgical Technique and Prosthetic Rehabilitation

Bilateral maxillary sinus lifts were performed with bone grafting, associating the biomaterial with leukocyte platelet-rich fibrin (L-PRF). For this procedure, red-capped tubes (n = 3) were used to make the membrane, using a protocol of 2700 rpm for 12 min, and white-capped tubes (n = 3) were used to make the graft-binding agent, using a protocol of 700 rpm for 4 min. The Intraspin centrifuge (Intralock International INC, Boca Raton, FL, USA) was used for this protocol. Both the white- and red-cap tubes were without internal chemical agents. The first two tubes of blood were collected and immediately placed into the centrifuge opposite each other to ensure the centrifuge was balanced correctly.

For the horizontal gain, the anterior region of the maxilla was drilled into the basal bone with a number 702 FG cross-cut conical surgical drill (American Burrs, Palhoça, Brazil) with an average of 10 perforations in each maxilla to enhance the graft’s vascularization process and potentiate the local healing response. After preparing the surgical bed, the region was filled by combining L-PRF with i-PRF to bind the synthetic biomaterial.

All cases were treated under local anesthesia and infiltrated with anesthetic mepivacaine hydrochloride 2% + epinephrine 1:100,000 (MEPIADRE, DFL, São Paulo, Brazil). An incision was made linearly and continuously over the alveolar bone crest of the maxilla, with two relaxing incisions in the region of the maxillary tuberosity. A mucoperiosteal detachment was then performed to expose the alveolar bone structure to be reconstructed. Two lateral bone windows were opened in the lateral wall of the sinus (20 mm long and 6 mm high) to access and detach the mucous membrane of the maxillary sinus and fill it with sticky bone associated with the bone graft.

Data were analyzed for success at the implant level, peri-implant soft tissue, prosthetics, and patient satisfaction. The criteria for success at the implant level were mobility, pain, radiolucency, and peri-implant bone loss (>1.5 mm), and for success at the peri-implant soft-tissue level, suppuration and bleeding. The criteria for success at the prosthetic level were technical complications/prosthetic maintenance, adequate function, and esthetics during the three years. The criteria at the patient satisfaction level were discomfort, satisfaction with appearance, and ability to chew/taste.

### 2.4. Post-Operative Evaluation

No complications were reported within the first five months following the intervention. After a minimum healing period of four months, grade 4 commercially pure titanium endosteal implants with a hydroxyapatite coating (S.I.N. Implant System, São Paulo, Brazil) were placed. The implants had average dimensions of 4.3 × 10 mm and were inserted with an average torque of 35 N. Reopening procedures were performed approximately five months after implantation, during which intermediaries were installed, and impressions were taken in the same session. Details regarding implant platform types, diameters, lengths, torques, surfaces, and models are provided in Table 1.

Following the reopening, Bränemark screw-retained fixed prostheses were installed using a prosthetic screw stabilization torque of 10 Ncm^−1^, following the manufacturer’s specifications. Patients were then enrolled in a three-year clinical and radiographic follow-up program, during which implant success criteria, peri-implant soft tissue health, prosthetic performance, and patient satisfaction were assessed.

Demographic and clinical variables, including age, gender, healing time after grafting (in months), number of implants placed, and type of antagonist, were also recorded for each patient (Table 2).

## 3. Results

The nine patients included in this case series were healthy, leucodermic adults requiring total maxillary rehabilitation, comprising seven women and two men, with an average age of 60 at the time of implant placement. All patients underwent systemic health assessments and received comprehensive pre- and post-operative care, including asepsis, anesthesia, and flap management. Diagnostic procedures involved clinical examinations, blood tests, medical evaluations, and panoramic radiographs to ensure accurate diagnosis and treatment planning.

The follow-up period ranged from 36 to 60 months after prosthesis installation, during which patient outcomes were systematically monitored (Table 2).

### Biomaterial

**Figure 2:**
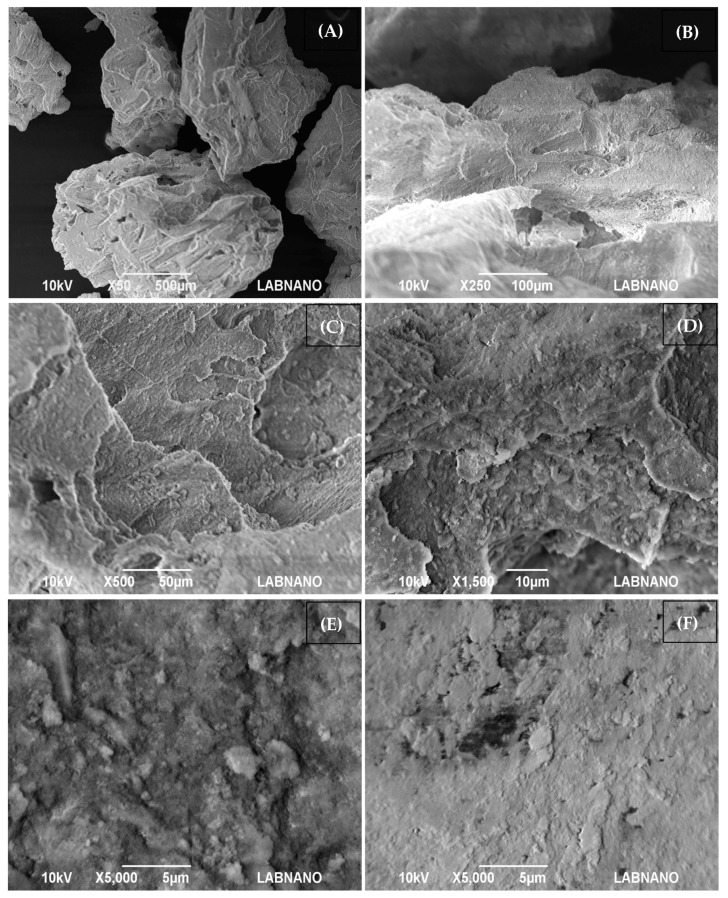
Scanning electron microscopy of Alobone^®^ Poros. (**A**) 50×; (**B**) 250×; (**C**) 500×; (**D**) 1500×; (**E**,**F**) 5000×. The size of the micrometer bar is indicated in each image. The micrographs show a highly porous architecture. The surface of the granules appears rough and irregular with small particles aggregated together to form larger, rough structures. This surface roughness can enhance osteoconductive, helping in the attachment of osteoblasts (bone-forming cells).

The Alobone^®^ Poros bone substitute biomaterial, a synthetic and radiopaque hydroxyapatite granule, exhibited particle sizes ranging from 250 to 1000 μm. Scanning electron microscopy (SEM) analysis revealed the material’s porous structure and a regular, homogeneous surface (Figure 2). Higher magnifications reveal finer details of the surface morphology, such as grooves, pits, or fractures, which can be important for cell adhesion and the eventual integration of the HA granules with surrounding bone tissue.

**Figure 3:**
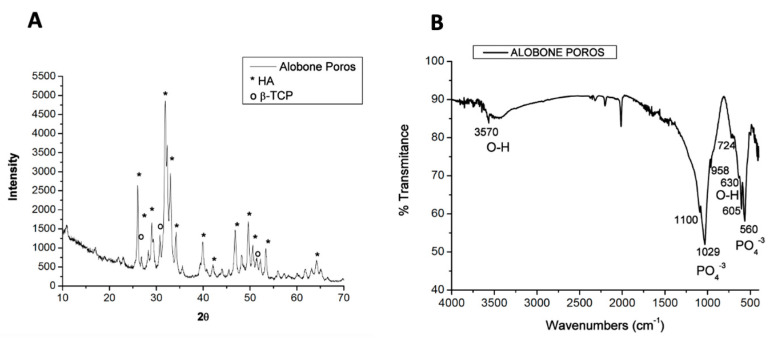
(**A**) X-ray diffractogram of Alobone Poros indicating hydroxyapatite (HA, *) and β-tricalcium phosphate (β-TCP, o) phases. (**B**) Fourier-transform infrared spectrum highlighting hydroxyl (OH^−^) and phosphate (PO_4_^−3^) groups.

X-ray diffraction (XRD) patterns for Alobone^®^ Poros indicated hydroxyapatite (HA) peaks with low crystallinity and β-tricalcium phosphate (β-TCP) peaks, also with low crystallinity (Figure 3A). Fourier-transform infrared (FTIR) spectroscopy identified bands between 1100 and 500 cm^−^¹, corresponding to the vibrational modes of (PO4)³^−^, and a band at 3570 cm^−^¹, representing the vibrational mode for (OH^−^) (Figure 3B).

**Figure 4:**
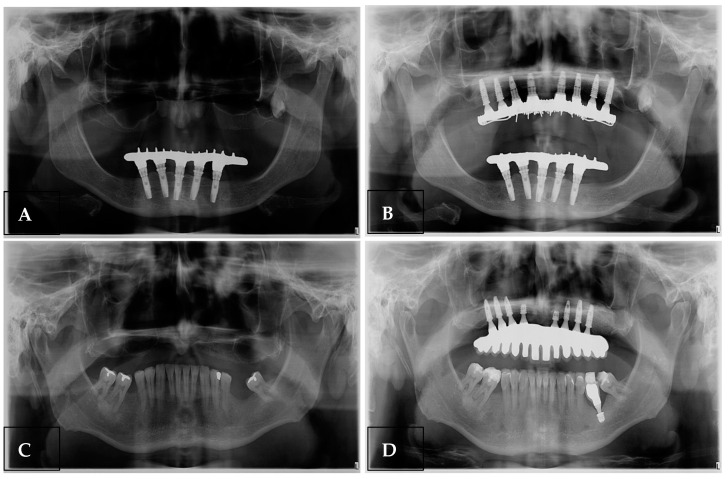
Panoramic radiographs of two clinical cases, (**A**,**C**) initial radiographs and (**B**,**D**) radiographs with the implants installed in the grafted areas 5 months after surgery.

Panoramic radiographs taken before and after the procedure demonstrated an increase in alveolar ridge height following grafting, which enabled the placement of eight implants in the atrophic maxillae of two patients (Figure 4).

All patients underwent clinical and radiographic evaluations three years post-procedure, meeting the success criteria for implant performance, peri-implant soft tissue health, prosthetic function, and patient satisfaction.

## 4. Discussion

Although various alternatives are available for maxillary and mandibular alveolar reconstruction to achieve optimal alveolar form, bone grafting procedures remain the most commonly used approach.

Alloplastic grafts combined with platelet concentrates are part of a group of techniques that have demonstrated favorable outcomes in the prosthetic rehabilitation of complex maxillary cases. The success of these therapies depends on several factors, including the patient’s systemic condition, surgical techniques, and prosthetic design. This study’s results indicate that using alloplastic biomaterials in conjunction with blood concentrates is a viable option for bone grafting and further rehabilitation of patients with severe maxillary atrophy.

Even though autogenous bone grafts are still considered the gold standard due to their faster healing times and superior integration between native and grafted bone, a recent systematic review found no significant differences in implant success rates among various bone augmentation materials, including autografts, xenografts, allografts, and alloplastic grafts. Patients generally preferred non-autogenous bone sources due to reduced hospital stays, less post-operative pain, and shorter recovery times. Another systematic review evaluated different biomaterials used in bone graft procedures for atrophic maxillae with resorbable membranes and particulate grafts. These procedures proved effective in regenerating bone, with the associated implants demonstrating high survival rates. However, the review noted a slightly higher rate of membrane exposure when cross-linked resorbable membranes were used [12].

The findings of this study demonstrate the long-term effectiveness of synthetic hydroxyapatite (HA) combined with leukocyte platelet-rich fibrin (L-PRF) in rehabilitating severely atrophic maxillae. These results contrast with those of Anis et al. (2024), who reported no significant clinical or statistical advantage of NanoBone^®^ (Artoss Inc., St Cloud, MN, USA) combined with PRF over PRF alone in the split-crest technique [13]. While Anis et al. [13] focused on horizontal bone augmentation and simultaneous implant placement with a shorter follow-up period, our study highlights the predictable long-term success of HA + L-PRF in more severe cases requiring both horizontal and vertical bone augmentation. The high implant success rate (98.5%) observed in our study supports the hypothesis that combining synthetic HA with L-PRF provides stable and durable outcomes, particularly in cases where simultaneous implant placement is not feasible. These differences underscore the importance of surgical technique, graft material properties, and follow-up duration in evaluating bone regeneration strategies.

Many authors have advocated for the use of titanium mesh as an adjunct to bone grafting, regardless of the graft’s origin. Although titanium mesh has a high incidence of exposure, these cases are typically resolved through minor interventions and rarely necessitate complete removal [14]. A recent systematic review demonstrated that titanium mesh supports bone repair due to its high resistance to deformation, preventing collapse. Consequently, titanium mesh has proven to be a predictable method for rehabilitating complex atrophic sites [15]. Applying resorbable membranes over titanium mesh reduces or delays mesh exposure.

Additionally, these membranes act as a barrier against oral contaminants, protecting the developing bone and promoting increased bone formation compared to cases where titanium mesh is exposed early and lacks membrane coverage. In this study, titanium meshes were not utilized. However, the approach successfully facilitated implant placement and enabled the loading of implant-supported total prosthesis.

The use of recombinant human bone morphogenetic protein-2 (rhBMP-2) in combination with grafts and titanium mesh has demonstrated success in treating atrophic maxillae [16] This approach creates an osteoinductive graft, enhancing vital bone formation and improving the healing of soft tissue and grafts. However, long-term data on craniofacial growth following rhBMP-2 application are still lacking, and its use in patients under 18 years of age remains off label [17].

Two primary approaches for addressing atrophic maxillae in dental implantology are the All-on-Four technique and bone grafting. The All-on-Four method involves placing four strategically positioned implants (two vertical and two angled) to support a full-arch prosthesis, often enabling same-day prosthesis loading and immediate function. This technique generally provides shorter recovery times and has a high patient satisfaction. In contrast, bone grafting involves augmenting deficient areas with additional bone, requiring several months for graft integration before implant placement. While this approach has a more extended recovery period and potential complications, such as graft rejection or infection, it offers long-term stability when proper healing occurs. The success of bone grafting depends on factors, such as graft site health and the quality of the graft material, whereas the All-on-Four technique’s success is attributed mainly to its immediate functionality and reduced number of implants required [18,19,20].

This study presents promising results; however, several limitations must be acknowledged. The relatively small sample size may limit the generalizability of the findings to larger populations. Additionally, this study’s retrospective nature restricted the ability to control variables as effectively as in a prospective, randomized controlled trial. Future research should focus on conducting more extensive, prospective, and controlled studies following standardized protocols to address these limitations. Such studies would provide more robust evidence to validate further the effectiveness of combining synthetic hydroxyapatite with leukocyte platelet-rich fibrin (L-PRF) in rehabilitating atrophic maxillae.

## 5. Conclusions

This case series highlights the effectiveness of combining synthetic hydroxyapatite grafting with L-PRF to rehabilitate severely atrophic maxillae. Over an average follow-up period of three years, a high implant success rate of 98.5% was achieved in nine patients, demonstrating excellent osseointegration and stable function across the 69 dental implants placed. This approach not only facilitated successful implant placement but also supported long-term prosthetic rehabilitation, contributing significantly to an improved quality of life for the patients.

The findings suggest that this combined technique represents a viable and predictable alternative to autogenous bone grafting for reconstructing atrophic maxillae. Moreover, it provides clinicians with an effective and reliable tool to address the challenges of implant placement in compromised alveolar ridges. Further research, including more extensive, prospective studies, is essential to confirm these results and refine the technique for broader clinical application.

## Figures and Tables

**Table 1:** Characteristics of implants installed according to the platform, diameter, length, torque, surface, and implant type.

Patients	Platform	Diameter	Length	Torque	Surface	Implant Type
1	CM	4.5	11.5	35 N	NanoHA	Epikut
2	CM	4.5	11.5	50 N	NanoHA	Strong
3	CM	4.3	13	45 N	NanoHA	Unitite
4	CM	3.8	11.5	40 N	NanoHA	Epikut
5	CM	4.3	8.5	20 N	NanoHA	Unitite
6	HE	3.75	10	20 N	NanoHA	Strong
7	CM	4.3	11.5	30 N	NanoHA	Unitite
8	CM	4.3	11.5	30 N	NanoHA	Unitite
9	CM	4.3	10	45 N	NanoHA	Unitite

**Table 2:** Demographic and implant site characteristics.

Patients	Gender	Age	Graft Surgery (Months)	Number of Implants	Antagonist
1	F	61	36	8	Natural teeth
2	F	71	60	8	Acrylic resin protocol
3	F	50	42	8	Natural teeth
4	F	50	36	8	Acrylic resin protocol
5	F	61	48	8	Acrylic resin protocol
6	F	70	48	8	Acrylic resin protocol
7	F	52	36	5	Natural teeth
8	M	63	42	8	Acrylic resin protocol
9	M	56	36	8	Ceramic protocol

## Data Availability

All data supporting the findings of this study are available within the paper.
